# Plasmon excitations with a semi-integer angular momentum

**DOI:** 10.1038/s41598-018-26196-9

**Published:** 2018-05-18

**Authors:** J. T. Mendonça, A. Serbeto, J. Vieira

**Affiliations:** 1 IPFN, Instituto Superior Técnico, Universidade de Lisboa, 1049-001 Lisboa, Portugal; 2 Instituto de Física, Universidade de São Paulo, São Paulo, SP 05508-090 Brazil; 3 Instituto de Física, Universidade Federal Fluminense, BR-24210-340 Niterói, RJ Brazil

## Abstract

We provide an explicit model for a spin-1/2 quasi-particle, based on the superposition of plasmon excitations in a quantum plasmas with intrinsic orbital angular momentum. Such quasi-particle solutions can show remarkable similarities with single electrons moving in vacuum: they have spin-1/2, a finite rest mass, and a quantum dispersion. We also show that these quasi-particle solutions satisfy a criterium of energy minimum.

## Introduction

Since its introduction in 1925 by Goudsmit and Uhlenbeck^1,2^, and the subsequent division of elementary particles into bosons and fermions, the spin plays a central role in modern physics^3,4^. It is well known that Dirac’s equation provides a physical model for the semi-integer (or fermionic) spin. On the other hand, the spin of bosons (such as photons and gravitons) can be explicitly described by the polarization properties of their respective fields.

Here we show that a spin-1/2 quasi-particle in a plasma can be explicitly described by using a superposition of Laguerre-Gauss modes, each one associated with an integer value of the angular momentum. We consider the case of plasmons, which are electrostatic excitations of a plasma. The electric field associated with these quasi-particles is purely longitudinal and parallel to the direction of propagation. This means that, from their polarization, we can conclude that the simplest plasmon solutions (which are plane wave solutions) have no intrinsic angular momentum, or no spin. However, it was recognized in recent years that electron plasma waves or plasmon solutions can exist with an integer intrinsic angular momentum. These are the so-called *twisted plasmons*^5,6^, which are the electrostatic equivalent of the twisted photons, traveling in vacuum with orbital angular momentum (OAM), as first considered by Allen and collaborators^7,8^. It was soon recognized that this photon OAM property could be divided into an intrinsic and an extrinsic part^9,10^, where the intrinsic part adds to the photon spin due to field polarization, and the extrinsic part is associated with the eventual bending of the photon beams, due to the inhomogeneities of the medium. The subtle differences and similarities between spin and intrinsic photon OAM were recently discussed by^11^.

The case of plasmons with OAM, to be considered here, is therefore much simpler than the case of photons, because they originally have no intrinsic (or polarization) spin. Therefore, the intrinsic value of their OAM can be directly associated with their spin. If we ignore the possible curvature of their propagation direction, then extrinsic OAM will be absent. It was shown by Berry^12^ that a superposition of OAM photon modes can lead to the formation of irrational light vortices in vacuum, with an arbitrary topological charge. Here we use a similar approach to show that a plasmon vortex can be built with a semi-integer topological charge, which can be seen as the physical realization of a spin-1/2 plasmon quasi-particle.

Apart from the semi-integer spin, these plasmon solutions also show other similarities with electrons moving in vacuum. For generality, we consider the case of a quantum plasma, from where the classical plasma case can be easily retrieved. In section II, we show the basic properties of plasmon (or electron plasma wave) solutions. The similarities with the single electron behavior in vacuum will be stressed. In particular, we show that an effective mass and an effective relativistic gamma-factor can be defined for plasmons. These quasi-particles satisfy a wave equation, formally analogous to a Klein-Gordon equation, which reduces to the Schrödinger equation in the appropriate (non-relativistic) limit.

In section III, we consider the twisted plasmon solutions with an intrinsic OAM, which can be directly associated with the plasmon spin. The basic solutions have an integer topological charge, and would correspond to the integer (or bosonic) spin case. They are described by Laguerre-Gauss (LG) field modes, very similar to those originally used for photons. The difference is that, in contrast with photons, plasmons have no spin polarization. The situation changes when we consider a particular superposition of LG field solutions, with a total semi-integer topological charge. This can then be used to describe a plasmon quasi-particle with spin-1/2. It will also be shown that the values of spin *s* = ±1/2 correspond to an energy minimum among all the possible states built with a superposition of LG modes. This provides a clear demonstration of the physical relevance of spin-1/2 quasi-particles: They minimize the energy. Finally, in section V, we state some conclusions.

## Plasmon Quasi-Particle

We consider high-frequency waves in a quantum plasma. Assuming the ions at rest, we describe the plasma behavior using the electron quantum fluid equations^13^

1 $$\frac{\partial n}{\partial t}+\nabla \cdot (n{\bf{v}})=0,\,\frac{\partial {\bf{v}}}{\partial t}+{\bf{v}}\cdot \nabla {\bf{v}}=\nabla (\frac{eV}{m}+{V}_{B})-\frac{\nabla P}{nm}.$$

They determine the evolution of the electron density *n* and mean velocity **v**, in the presence of an electrostatic potential *V*. This potential, and the Bohm potential (also sometimes referred as the quantum pressure) *V*_*B*_ are determined by

2 $${\nabla }^{2}V=\frac{e}{{\epsilon }_{0}}(n-{n}_{0}),\,{V}_{B}=\frac{{\hslash }^{2}}{2{m}^{2}}\frac{{\nabla }^{2}\sqrt{n}}{\sqrt{n}}.$$

We assume small perturbations $\tilde{n}=n-{n}_{0}$ around the equilibrium plasma density *n*_0_, and neglect the nonlinear terms. We also use for the pressure a simple equation of state satisfying $\nabla P=m{v}_{F}^{2}\nabla \tilde{n}$, where *v*_*F*_ is the electron Fermi velocity. Assuming an uniform and infinite medium, we can easily get from the above equations a wave equation of the form

3 $$(\frac{{\partial }^{2}}{\partial {t}^{2}}+{\omega }_{p}^{2}-{v}_{F}^{2}{\nabla }^{2}+{\beta }^{2}{\nabla }^{4})\,\tilde{n}=0,$$

where *ω*_*p*_ is the electron plasma frequency, and the perturbed Bohm potential ${\tilde{V}}_{B}$ and quantum factor *β*^2^ are determined by

4 $${\tilde{V}}_{B}={\beta }^{2}\frac{{\nabla }^{2}\tilde{n}}{{n}_{0}},\,{\beta }^{2}=\frac{{\hslash }^{2}}{4{m}^{2}}.$$

In order to solve this equation, we assume simple wave solutions of the form $\tilde{n}={n}_{k}\,\exp (ikz-i\omega t)$, describing propagation along the arbitrary Oz-direction, with frequency *ω* and wavenumber *k*. We also assume that the wave amplitude *n*_*k*_ slowly varies along propagation ($\partial {n}_{k}/\partial z\ll k{n}_{k}$), and it also slowly varies in the transverse direction ${{\rm{r}}}_{\perp }$, in such a way that the Helmholtz equation is satisfied

5 $$({\nabla }^{2}+{k}^{2})\,\tilde{n}=0.$$

Replacing this in eq. (3), we obtain the well known dispersion relation for electron plasma waves in a quantum plasma

6 $${\omega }^{2}={\omega }_{p}^{2}+{v}_{F}^{2}{k}^{2}+{\beta }^{2}{k}^{4}.$$

Following the usual concepts of plasma turbulence, we can say that an arbitrary spectrum of electron plasma waves is equivalent to an ensemble of quasi-particles, or plasmons. Defining the plasmon energy as ${\epsilon }_{k}=\hslash {\omega }_{k}$, and introducing the plasmon effective mass *m*_*f*_, we can obtain from the above dispersion relation the following relation between the energy and the plasmon momentum *p* = *ħk*, as

7 $${\epsilon }_{k}=\sqrt{{W}^{2}+{\beta }^{2}{p}^{4}/{\hslash }^{2}},$$

where *W* is formally identical to the plasmon energy in a classical plasma (apart from the replacement of the thermal velocity by *v*_*F*_), as defined by

8 $$W=\sqrt{{m}_{f}^{2}{v}_{F}^{4}+{p}^{2}{v}_{F}^{2}}\equiv {m}_{f}{\gamma }_{f}{v}_{F}^{2}.$$

The plasmon effective mass *m*_*f*_ and the plasmon effective relativistic factor *γ*_*f*_, are defined as

9 $${m}_{f}=\frac{\hslash {\omega }_{p}}{{v}_{F}^{2}},\,{\gamma }_{f}=\sqrt{1+\frac{{p}^{2}}{{p}_{F}^{2}}},$$

with ${p}_{F}={m}_{f}{v}_{F}$. We can also write the effective relativistic factor in a alternative way, as

10 $${\gamma }_{f}=\sqrt{1+\frac{{\hslash }^{2}{k}^{2}}{{m}_{f}^{2}{v}_{F}^{2}}}=\sqrt{1+\frac{{k}^{2}{v}_{F}^{2}}{{\omega }_{p}^{2}}}.$$

We can see from the above equations that the plasmon dynamics in a quantum plasma looks formally identical to that of a single massive particle (such as an electron) moving in vacuum. Here, the Fermi velocity *v*_*F*_ plays the role of the speed of light in vacuum *c*, as an asymptotic velocity limit.

In a classical plasma, *v*_*F*_ would be replaced by the thermal velocity ${S}_{e}=\sqrt{3T/m}$, where *T* is the electron temperature. We can see that the quantum plasma corrections, associated with the term in *β*^2^, shown in eq. (7), introduce a small deviation from this interesting analogy with vacuum electrodynamics. Such formal analogies and, in particular, the existence of an effective plasmon mass *m*_*f*_, had already been considered before for a classical plasma, when *v*_*F*_ is replaced by the thermal velocity and the *β*^2^ corrections are absent^14^. We should however notice that coupling between the particle spin and the electromagnetic spin, usually present in quantum electrodynamics, is missing for plasmon quasi-particles.

The analogy between a plasmon quasi-particle and an electron in vacuum can be further explored by returning to the wave equation (3). It is known that, due to electron Landau damping, the plasmon frequencies are always of the order of *ω*_*p*_. This means that a solution of this equation can also be written in the form

11 $$\tilde{n}({\bf{r}},t)={\rm{\Phi }}({\bf{r}},t)\,\exp (\,-\,i{\omega }_{p}t),$$

where Φ ≡ Φ(**r**, *t*) contains a small deviation of the wave frequency with respect to the plasma frequency, such that we can take $|\partial {\rm{\Phi }}/\partial t|\ll |\omega {\rm{\Phi }}|$. Replacing this in eq. (3), and rearranging terms, we can easily derive for Φ, the envelope equation

12 $$(\,-2i{\omega }_{p}\frac{\partial }{\partial t}-{v}_{F}^{2}{\nabla }^{2}+{\beta }^{2}{\nabla }^{4})\,{\rm{\Phi }}=0,$$

Multiplying by *ħ*, and introducing the above definition of the plasmon effective mass, we can then rewrite this in the standard form of a Schrödinger equation, as

13 $$i\hslash \frac{\partial {\rm{\Phi }}}{\partial t}=(\,-\frac{{\hslash }^{2}}{2{m}_{f}}{\nabla }^{2}+{K}_{Q})\,{\rm{\Phi }},$$

with

14 $${K}_{Q}={\beta }^{2}\frac{{\hslash }^{2}{\nabla }^{4}}{2{m}_{f}{v}_{F}^{2}}$$

We recognize on the r.h.s. of eq. (13) the usual kinetic energy operator, written in therms of the effective plasmon mass *m*_*f*_, and a quantum correction *K*_*Q*_, which results from the Bohm potential and represents the quantum plasma response. This clearly shows that a plasmon can be described as a quantum quasi-particle, satisfying a Schrödinger equation, written in the usual form. Such a description is valid even in the case of a classical plasma, where the additional term ${K}_{Q}$ is absent. The derivation of this Schrödinger equation is not surprising, given the fact that our initial wave equation (3) is formally identical to a Klein-Gordon equation. The small deviation of *ω* with respect to *ω*_*p*_ can therefore be seen as a small kinetic energy with respect to the rest mass (energy) of the plasmon quasi-particle. This means that such quasi-particles are always in a kind of non-relativistic regime. This has nothing to do with the plasma regime itself, which was considered from the beginning as (quantum but) non-relativistic, as described by eq. (1).

## Twisted Plasmons

We now examine the spatial structure of the plasmon modes, and discuss the possible solutions compatible with the assumed Helmholtz equation (5). In homogeneous and infinite medium, we can of course use plane wave solutions such that the density amplitude perturbation is the same in any point of the perpendicular planes, corresponding to *n*_*k*_ = *const*. But here we focus on pulsed or beam wave solutions, finite in the transverse direction, such that ${n}_{k}\equiv {n}_{k}({{\bf{r}}}_{\perp },z)$ is also assumed to vary slowly along the propagation direction *Oz*. In this case, we can derive from (5) a *paraxial equation*, of the form

15 $$({\nabla }_{\perp }^{2}+2ik\frac{\partial }{\partial z})\,{n}_{k}=0.$$

It is well known^15^ that the general solution of this equation can be represented by a superposition of orthogonal Laguerre-Gauss (LG) modes, *F*_*lp*_(**r**), defined as

16 $${F}_{lp}(r,\theta ,z)={c}_{lp}{X}^{|l|/2}{L}_{p}^{|l|}(X)\,\exp (il\theta -X/2)$$

where ${L}_{p}^{l}$ are the associated Laguerre polynomials of argument *X* = *r*^2^/*w*^2^(*z*), where *w*(*z*) defines the (slowly varying) transverse size of the wave beam. The normalization factors are defined as

17 $${c}_{lp}=\frac{1}{2\sqrt{\pi }}\,{[\frac{(l+p)!}{p!}]}^{1/2},$$

in order to satisfy the orthonormality relations

18 $$\langle {F}_{lp}|{F}_{l^{\prime} p^{\prime} }\rangle \equiv {\int }_{0}^{\infty }\,rdr\,{\int }_{0}^{2\pi }\,d\theta \,{F}_{lp}^{\ast }(r,\theta ,z){F}_{l^{\prime} p^{\prime} }(r,\theta ,z)={\delta }_{pp^{\prime} }{\delta }_{ll^{\prime} }.$$

This means that a plasmon beam solution, satisfying the dispersion relation (6) and propagating along the *z*-axis, can be generally described as

19 $$\tilde{n}({\bf{r}},t)={n}_{k}{e}^{i\phi (z,t)}=\sum _{l,p}\,{u}_{lp}{F}_{lp}(r,\theta ,z)\,\exp [i\phi (z,t)],$$

where *u*_*lp*_ are the LG mode amplitudes and *φ*(*z*, *t*) = (*kz* − *ωt*) is the phase function. Notice that such a beam solution is compatible with the above plasmon dispersion relation, which is usually associated with simple plane waves. Such a validity is guaranteed by the use of the Helmholtz equation (5), which establishes a link between the dispersion relation and the paraxial equation.

Next, we demonstrate that, by conveniently choosing the mode coefficients *u*_*lp*_, it is possible to built a plasmon solution with an intrinsic angular momentum *s* = ±1/2. But, in order to keep our discussion on more general grounds, we consider the case where the above expression will reduce to a solution of the form

20 $$\tilde{n}({\bf{r}},t)=R(r,z)\,\exp [i\mu \theta +i\phi (z,t)],$$

where *μ* is a real number. For convenience, we define *μ* inside the interval (−1, +1). Using the orthogonality condition (18), we can see that the mode coefficients in eq. (19) are *u*_*lp*_ = 〈*F*_*lp*_|*n*_*k*_〉. On the other hand, multiplying eq. (20) by *F*_*lp*_ and integrating over (*r*, *θ*), we obtain 〈*F*_*lp*_|*n*_*k*_〉 = 〈*F*_*lp*_|*Re*^*iμθ*^〉. This allows us to choose

21 $${u}_{lp}=\langle {F}_{lp}(r,\theta ,z)|R(r,z)\,\exp (i\mu \theta )\rangle .$$

In order to derive an explicit expression for these quantities, we rewrite this expression in an alternative and more explicit form, as

22 $${u}_{lp}={c}_{lp}\,{I}_{r}(l,p){I}_{\theta }(\mu ,l),$$

with the integrals

23 $${I}_{r}(l,p)={\int }_{0}^{\infty }\,R(r,z){X}^{|l|/2}{L}_{p}^{|l|}(X){e}^{-X/2}rdr,$$

and

24 $${I}_{\theta }(\mu ,l)={\int }_{0}^{2\pi }\,\exp \,[i(\mu -l)\theta ]\,d\theta .$$

At this point, it is useful to define the auxiliary function *f*(*θ*) = exp(*iμθ*), and to use its Fourier series expansion

25 $$f(\theta )=\sum _{n=-\infty }^{\infty }\,{g}_{n}{e}^{in\theta },\,{g}_{n}=\frac{1}{2\pi }\,{\int }_{0}^{2\pi }\,f(\theta ){e}^{-in\theta }d\theta .$$

The Fourier coefficients can easily be calculated, as

26 $${g}_{n}=\frac{1}{2\pi i}\frac{1}{(\mu -n)}\,[{e}^{2\pi i(\mu -n)}-1]=\frac{1}{\pi }\frac{\exp (i\mu \pi )}{(\mu -n)}\,\sin (\mu \pi ).$$

We now focus on the particular case *μ* = *s* = ±1/2. Comparison with the general case will be considered later. Noting that, for *s* = ±1/2, we have sin(*sπ*) = 2*s*, we can use the above results to derive the identity

27 $${e}^{is\theta }=\frac{2s}{\pi }{e}^{is\pi }\,\sum _{n=-\infty }^{\infty }\,\frac{{e}^{in\theta }}{(s-n)}.$$

A similar approach has been already use by Berry^12^ to describe optical vortices with arbitrary topological charge *μ*. Here we focus on the particular case of *μ* = *s*, which is specially important, as shown below. Introducing this result in the integral of eq. (24), we obtain

28 $${I}_{\theta }(s,l)=\sum _{n=-{\rm{\infty }}}^{{\rm{\infty }}}\,{g}_{n}\,{\int }_{0}^{2\pi }\,\exp \,[i(n-l)\theta ]\,d\theta =2\pi {g}_{l}.$$

Now, replacing this in eq. (22) we get an expression for the LG mode coefficients of the form

29 $${u}_{lp}=4s{c}_{lp}\frac{{e}^{is\pi }}{(s-l)}{I}_{r}(l,p),$$

**Figure 1:**
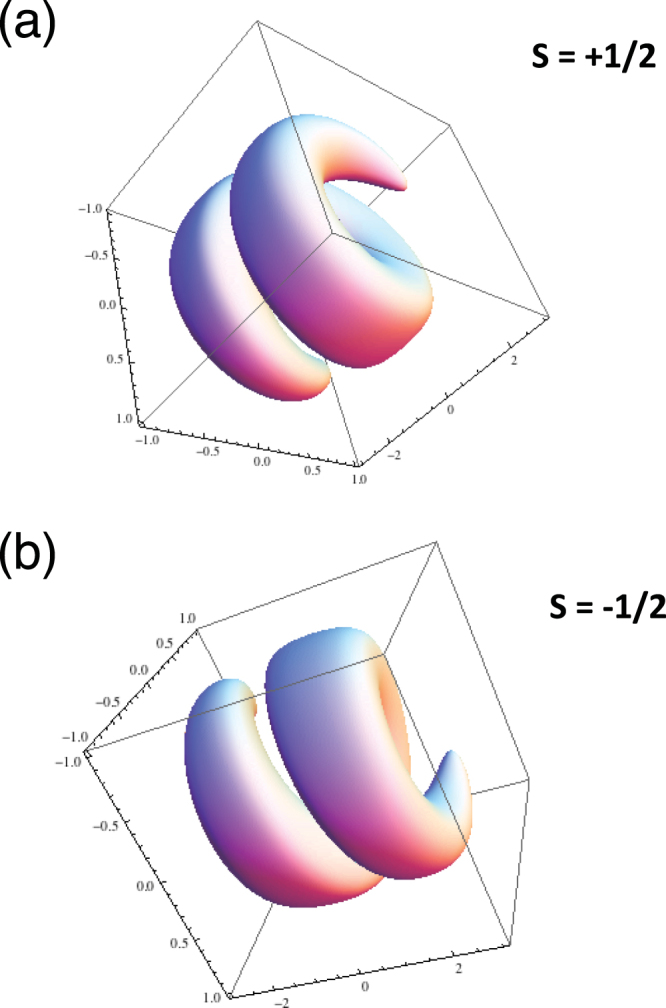
Electron density perturbations associated with twisted plasmon solutions with semi-integer topological charge *s* = ±1/2.

We can see that the explicit value of the quantities *u*_*lp*_ allowing us to built an electrostatic vortex with topological charge *s* will only depend on the transverse shape of the plasmon mode, *R*(*r*, *z*). The two plasmon states are illustrated in Fig. 1.

Let us now consider another approach, which allows us to define the transverse shape *a posteriori*. For that purpose, we return to eq. (19) and rewrite it in the form

30 $${n}_{k}=\sum _{l,p}\,{u}_{lp}{R}_{lp}(r,z)\,\exp (il\theta ),$$

where the new radial profile function *R*_*lp*_(*r*, *z*), which is independent of the poloidal variable *θ*, is related to the LG mode functions by the obvious expression

31 $${R}_{lp}(r,z)={F}_{lp}(r,\theta ,z)\,\exp (\,-\,il\theta )$$

We now take the complex conjugate of eq. (27) and write the new identity

32 $$1=\frac{2s}{\pi }{e}^{is(\theta -\pi )}\,\sum _{n=-\infty }^{\infty }\,\frac{{e}^{-in\theta }}{(s-n)}.$$

Multiplying this unit factor to the r.h.s. of eq. (30), we obtain

33 $${n}_{k}={R}_{s}(r,z)\,\exp (is\theta ),$$

with

34 $${R}_{s}(r,z)=\frac{2s}{\pi }{e}^{-is\pi }\,\sum _{lp}\,\sum _{n}\,\frac{{e}^{i(l-n)\theta }}{(s-n)}{u}_{lp}{R}_{lp}(r,z).$$

But, it should be noticed that this quantity is by definition independent of *θ*, which means that we need to assume that *l* = *n*. If we further consider the simplest possible radial profile, which is associated with the radial index *p* = 0, we are then reduced to

35 $${R}_{s}(r,z)=\frac{2s}{\pi }{e}^{-is\pi }\,\sum _{l}\,\frac{{u}_{l0}}{(s-l)}{R}_{l0}(r,z).$$

This expression allows us to define the plasmon radial profile in a simpler and more explicit way.

At this point, we should consider the energy of these twisted wave solutions. For completeness, we go back to the case of a generic topological charge *μ* ≠ *s*. It should be noticed that the energy associated with the twisted plasmons with angular momentum (or topological charge) *μ*, can be generally determined by eq. (19) as

36 $$|\tilde{n}{|}^{2}=\sum _{lp}\,|{u}_{lp}{|}^{2},$$

where integration over the transverse plane (*r*, *θ*) was assumed, and orthonormality of the LG mode functions *F*_*lp*_(*r*, *θ*, *z*) was considered. Using eq. (29), this can also be written as

37 $$|\tilde{n}{|}^{2}=4\,\sum _{lp}\,\frac{|{c}_{lp}{|}^{2}}{{(\mu -l)}^{2}}{I}_{r}{(l,p)}^{2}\,{\sin }^{2}(\mu \pi ),$$

This expression shows that the various contributions from the different LG modes decay with *l*^−2^, which allows us to retain just the first dominant modes with lower angular momentum. If we also assume that the coefficients in the numerator are all of the same order of magnitude, we can write a simplified expression for the plasmon energy as

38 $$|\tilde{n}{|}^{2}\simeq 4f(s),\,f(s)=|{c}_{10}{|}^{2}{I}_{r}{(1,0)}^{2}\,\sum _{l=0,\pm 1,\pm 2}\,\frac{{\sin }^{2}(\mu \pi )}{{(\mu -l)}^{2}},$$

**Figure 2:**
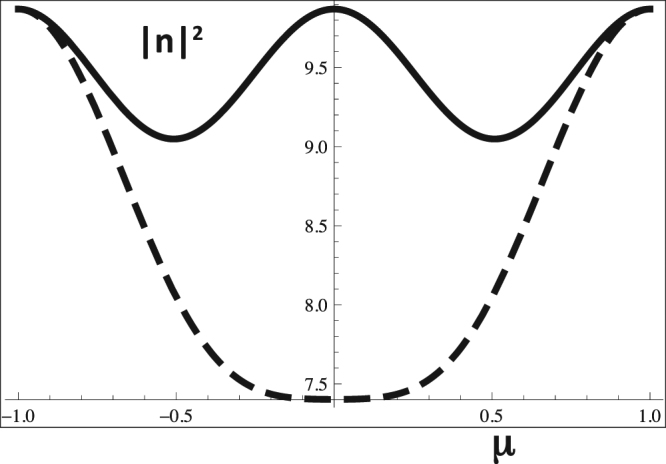
Approximate value of the total energy associated (a.u.), for an arbitrary value of the topological charge *μ*, varying in the interval (−1, 1). The energy shows two minima, at *μ* ≡ *s* = ±1/2. When we reduce the energy of the LG mode *l* = 0, from *μ* = −1 to 1, these energy minima disappear, as shown in the dashed curve (for *μ* = 0.75).

In principle, the quantity *μ*, could take arbitrary values inside the interval (−1, 1). But, it can easily be shown that the energy of the twisted wave solutions has a minimum for *μ* = *s*. This allows us to understand the particular relevance of the spin-*s* = ±1/2 case: because it minimizes the energy. This is illustrated in Fig. 2. This energy minimum is absent in the integer case, when *s* is replaced by *l* and the plasmon wave packet reduces to a single LG mode.

## Conclusions

In conclusion, we have shown that plasmons behave in a way that shows strong similarities with the behavior of a relativistic particle in vacuum. In particular, an effective mass and an effective relativistic *γ*-factor can be defined for such quasi-particles. The Fermi velocity (or the thermal velocity, in a classical plasma) replaces the velocity of light in vacuum *c*, as the maximum possible speed.

We have also shown that the spin-1/2 plasmons can be built by a superposition of twisted LG modes. Furthermore, the envelope equation for the plasmon field can be written in the standard form of a Schrödinger equation. This means that, in many respects, the spin-1/2 plasmon behavior shows very strong qualitative similarities with the electron behavior in vacuum. Quantum plasma contributions to the plasmon dispersion were also retained. They introduce small deviations to this analogy. Furthermore, it was shown that the case *s* = ±1/2 corresponds to an energy minimum among all the possible superpositions of the LG plasmon modes. This could give special relevance to the semi-integer spin case with respect to all other possible mode configurations.

The interest of such analogies is that they point to the existence of a new type of plasmonic structure, never considered before in the literature. In recent years, we have learned that intense laser pulses could excite non-trivial forms of electrostatic waves, such as donut-shape wakefields^16^. In the present paper, we show that spin-1/2 plasmons could also be excited in a plasma. This could eventually lead to new experimental studies. The next step is to understand the nonlinear regime, and the resulting wave-particle processes.

Finally, we should not forget the fundamental differences between an elementary particle in vacuum (the electron) and a quasi-particle in a medium (the plasmon). In particular, in contrast with the electrons, there is no intrinsic magnetic moment associated with these plasmon states, at least in the linear approximation. The magnetic properties of plasmons, in the nonlinear regime, and the possible use of laser-plasma interactions to excite spin-1/2 plasmon structures, will be addressed in the future.
